# RAS testing and cetuximab treatment for metastatic colorectal cancer: a cost-effectiveness analysis in a setting with limited health resources

**DOI:** 10.18632/oncotarget.17029

**Published:** 2017-04-11

**Authors:** Bin Wu, Yuan Yao, Ke Zhang, Xuezhen Ma

**Affiliations:** ^1^ Medical Decision and Economic Group, Department of Pharmacy, Ren Ji Hospital, South Campus, School of Medicine, Shanghai Jiaotong University, Shanghai, P.R. China; ^2^ Department of Laboratory, The Affiliated Hospital of Qingdao University, Qingdao, Shandong, P.R. China; ^3^ Department of Medical Oncology, Qingdao Commercial Worker‘s Hospital, Qingdao, Shandong, P.R. China; ^4^ Department of Medical Oncology, Qingdao Central Hospital, The Second Affiliated Hospital of Qingdao University Medical College, Qingdao, Shandong, P.R. China

**Keywords:** colorectal cancer, cost-effectiveness, cetuximab, gene mutation

## Abstract

**PURPOSE:**

To test the cost-effectiveness of cetuximab plus irinotecan, fluorouracil, and leucovorin (FOLFIRI) as first-line treatment in patients with metastatic colorectal cancer (mCRC) from a Chinese medical insurance perspective.

**RESULTS:**

Baseline analysis showed that the addition of cetuximab increased quality-adjusted life-years (QALYs) by 0.63, an increase of $17,086 relative to FOLFIRI chemotherapy, resulting in an incremental cost-effectiveness ratio (ICER) of $27,145/QALY. When the patient assistance program (PAP) was available, the ICER decreased to $14,049/QALY, which indicated that the cetuximab is cost-effective at a willingness-to-pay threshold of China ($22,200/QALY). One-way sensitivity analyses showed that the median overall survival time for the cetuximab was the most influential parameter.

**METHODS:**

A Markov model by incorporating clinical, utility and cost data was developed to evaluate the economic outcome of cetuximab in mCRC. The lifetime horizon was used, and sensitivity analyses were carried out to test the robustness of the model results. The impact of PAP was also evaluated in scenario analyses.

**CONCLUSIONS:**

RAS testing with cetuximab treatment is likely to be cost-effective for patients with mCRC when PAP is available in China.

## INTRODUCTION

Colorectal cancer (CRC) is one of the most common human malignancies and a leading cause of cancer-related death worldwide in developed countries [1]. The age-standardized incidence of colorectal cancer in China is 16.9 per 100,000 in males and 11.6 per 100,000 in females, and the age-standardized mortality is 9.0 per 100,000 in males and 6.1 per 100,000 in females [2]. Nearly 15% of CRC patients are diagnosed with metastatic disease at the time of diagnosis, and nearly half of these patients develop metastases during the course of their disease [3]. Despite the improvements in diagnosis and treatment, metastatic colorectal cancer (mCRC) remains an incurable disease with a 2-year median overall survival time [4]. Clearly, new treatments for mCRC are necessary to improve the poor clinical outcomes.

Over the past decade, the clinical benefits of monoclonal antibodies to epidermal growth factor receptor (EGFR), including cetuximab and panitumumab, combined with chemotherapy or monotherapy in mCRC patients have been shown [5]. Cetuximab was approved by the Chinese Food and Drug Administration in 2006. However, the response to cetuximab is influenced by a number of factors, the best known being KRAS gene status [6]. Previous pivotal studies have indicated that patients with KRAS wild-type mCRC will obtain a significant improvement in overall survival (OS), progression-free survival (PFS) and overall response rate (ORR) by adding cetuximab to standard chemotherapy. However, those with KRAS mutations are more likely to benefit from standard chemotherapy alone [7–9]. Thus, clinical guidelines have recommended cetuximab for the treatment of patients with EGFR-expressing, KRAS wild-type mCRC [10]. Therefore, KRAS mutation screening is an important component of the diagnostic plan [11]. Because of the resources required for mutation screening and cetuximab treatment, financial concerns might limit this evaluation. Economic analyses have indicated that cetuximab offers good value-for-money in patients with mCRC in developed countries [11–20]. However, these results might not be applicable for decision making in China because of the limited health resources in China and Western regions.

In regard to the issues mentioned above, the current goal was to examine the outcomes of KRAS screening followed by targeted first-line cetuximab treatment for mCRC from the perspective of Chinese payers.

## RESULTS

### Base-case analysis

The results of a base-case analysis with a 10-year time horizon, as well as economic and health outcomes estimated by the model, are shown in Table 1. For patients with advanced mCRC, the cetuximab regimen yielded an increase of 0.149 progression-free life-years (LYs), 0.73 overall LYs, or 0.63 quality-adjusted life-years (QALYs) in comparison with the chemotherapy regimen. The incremental direct medical cost amounted to $8,843 and $17,086 with and without a patient assistance program (PAP) over the 10-year period, respectively. The incremental cost-effectiveness ratio (ICER) for adding cetuximab to irinotecan, fluorouracil, and leucovorin (FOLFIRI) chemotherapy was $14,049 and $27,145 per QALY saved with and without PAP, respectively.

**Table 1:** Summary of cost ($) and outcome results from a base-case analysis

Regimen	Cost	Progression-free LYs	Overall LYs	QALYs	Incremental cost per QALY*	Incremental cost per LY*
FOLFIRI (control regimen)	30,668	0.795	2.066	0.963		
Cetuximab with PAP	39,511	0.944	2.796	1.593	14,049	12,107
Cetuximab without PAP	47,754	0.944	2.796	1.593	27,145	23,393

* Compared to a control regimen.

### Sensitivity analysis

**Figure 1:**
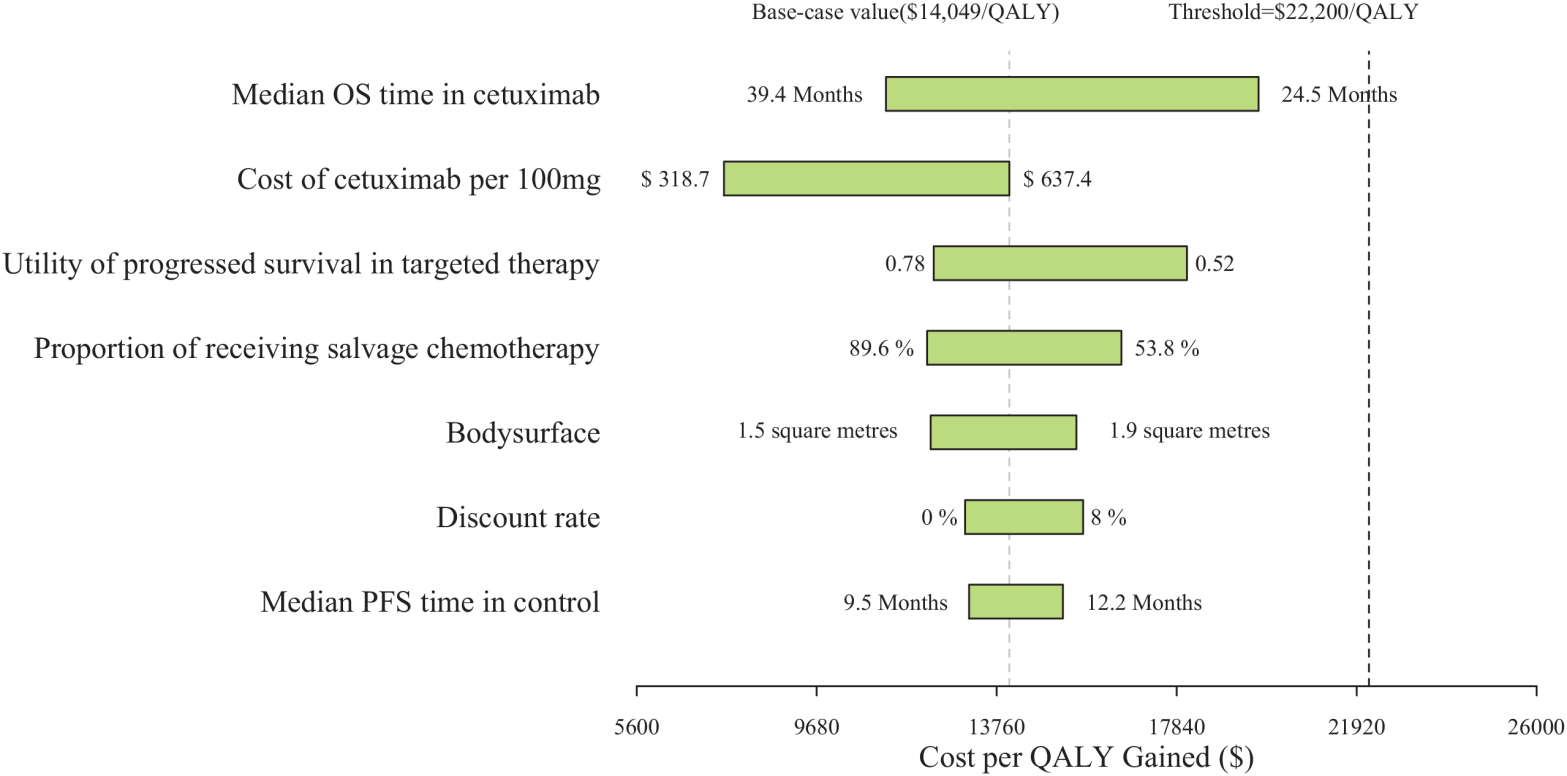
One-way sensitivity analysis for the cetuximab regimen using PAP versus the control regimen PFS: progression-free survival; OS, overall survival; RAS: rat sarcoma viral oncogene homolog; QALY: quality-adjusted life-year.

One-way sensitivity analysis revealed the most sensitive model parameters (Figure 1). The most sensitive parameters in the cetuximab regimen using PAP compared to the control included median OS time and cost of cetuximab. Other parameters, such as the cost and probability of managing severe adverse events (SAEs), showed moderate or little impact on the model’s outcome.

**Figure 2:**
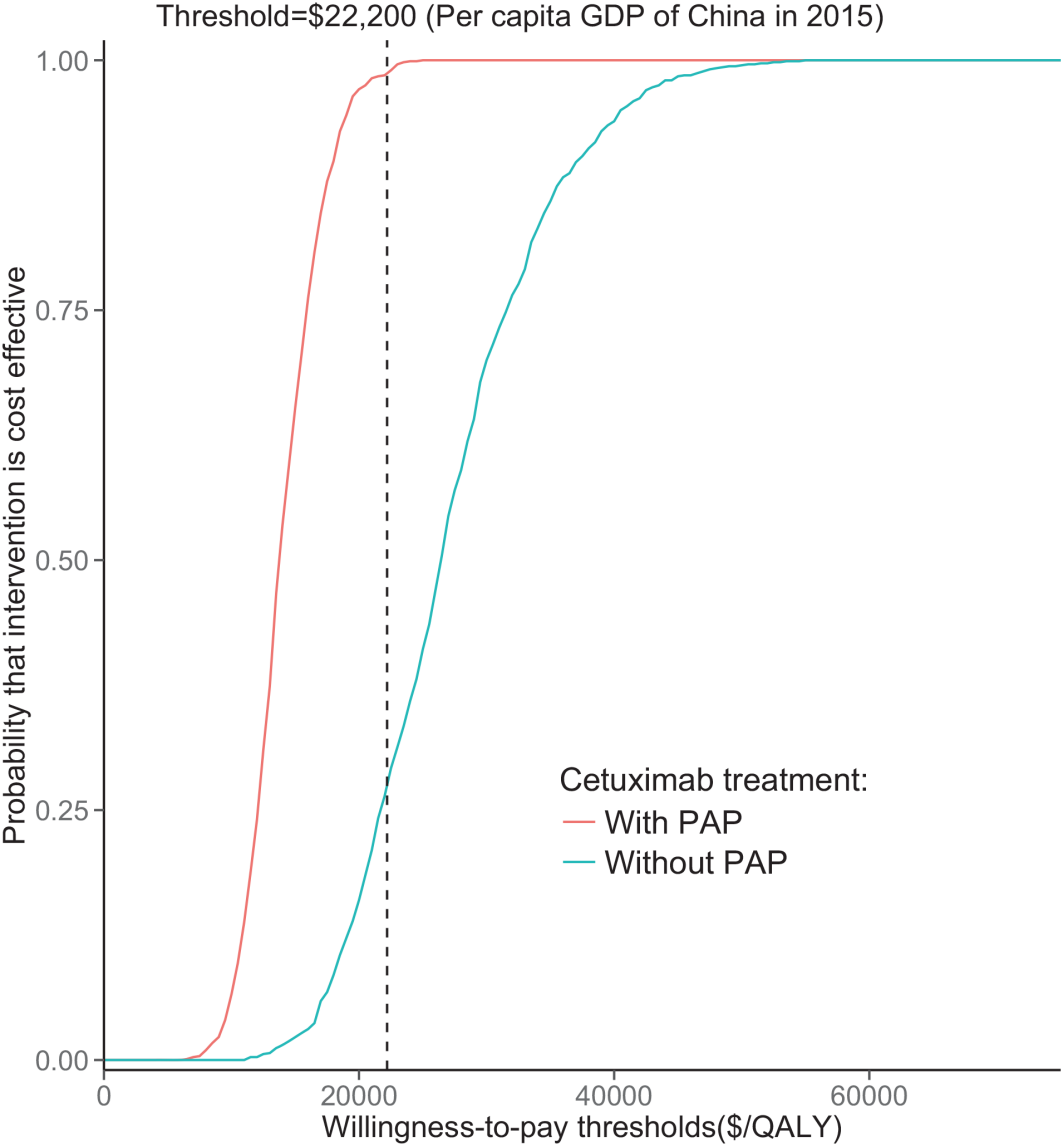
Acceptability curves comparing the cost-effectiveness of the cetuximab regimen with PAP or without PAP versus the control regimen The y-axis indicates the probability that a strategy is cost-effective across the willingness to pay per QALY gained (x-axis). The vertical dashed line represent the thresholds for China. QALY: quality-adjusted life-year.

The results of the probabilistic sensitivity analyses (PSA) are shown via cost-effectiveness acceptability curves (Figure 2). With PAP, the proportions of simulations being cost-effective for cetuximab were nearly 90% in comparison with the control regimen at a cost-effectiveness threshold of US $22,000. When no PAP was available, the control regimen achieved 75% likelihood of cost-effectiveness.

## DISCUSSION

**Figure 3:**
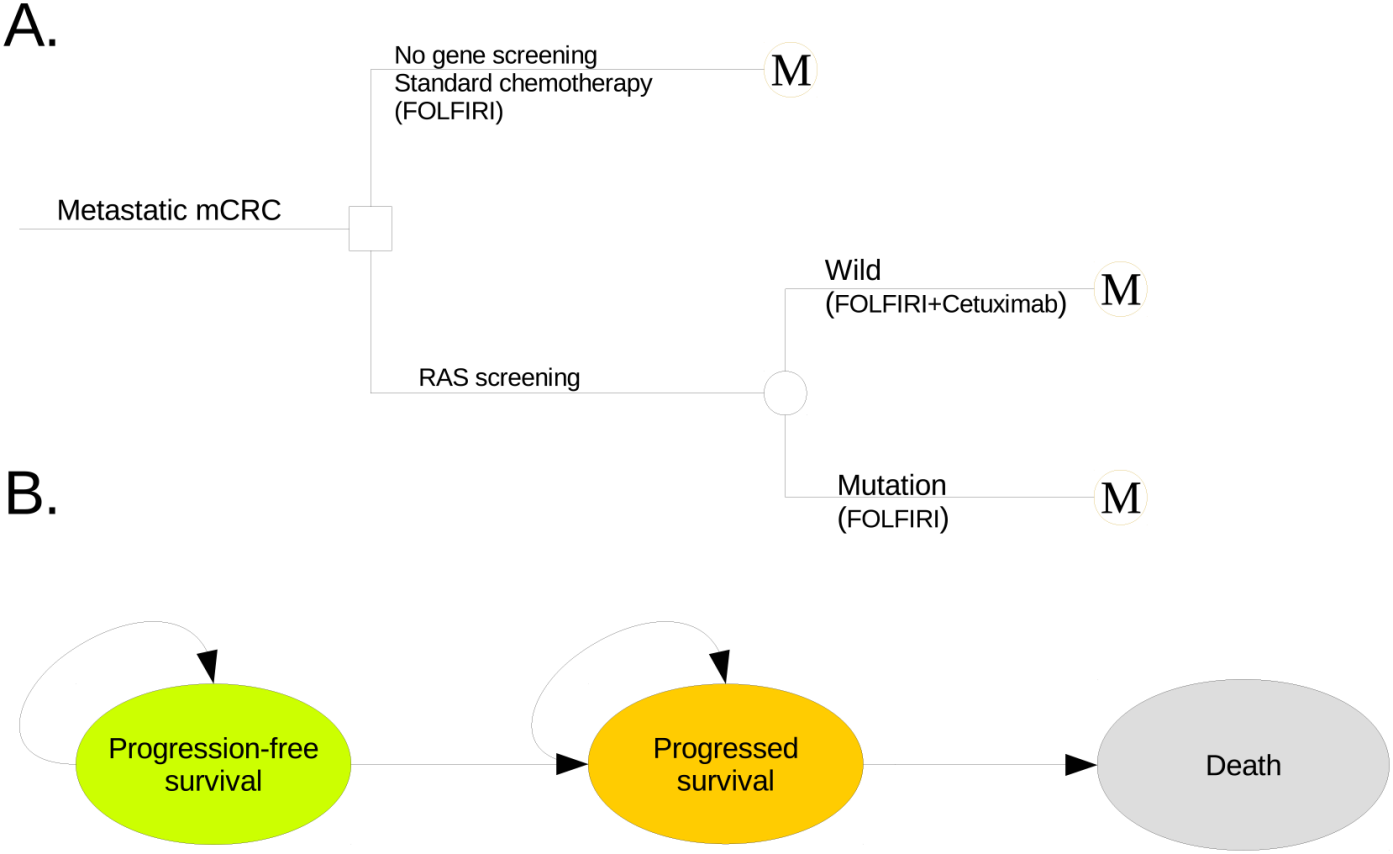
The schematics of the decision tree (A) and the Markov state transition model (B) CRC: colorectal cancer.

Using a Markov analysis model to assess wild-type RAS mCRC, we found that the 10-year ICER for adding cetuximab to traditional chemotherapy was generally unfavorable, at $61,746 per QALY gained. The ratios were largely attributable to the higher cost associated with the acquisition of cetuximab, whereas other costs, such as RAS mutation testing and management of progressed disease, had little impact. This result was robust based on the results of PSA. For cetuximab PAP, cetuximab treatment with RAS testing for patients with wild-type RAS mCRC might be the most cost-effective option because their ICERs are lower than the threshold, and the probability of cost-effectiveness reaches 90% at a threshold of $22,000 (Figure 3). These results suggest that cetuximab might be cost-effective in the PAP setting, which was supported by the sensitivity analysis. Furthermore, the cost of cetuximab is a sensitive parameter, as shown by a one-way sensitivity analysis. Other studies have also found that the ICER of cetuximab compared to that of other treatments for mCRC patients is high and is sensitive to drug costs [11–20].

To our knowledge, the current report is the first economic analysis evaluating cetuximab for the treatment of wild-type RAS mCRC patients in a representative setting with limited health resources. The pharmacoeconomic results indicate that RAS mutation testing and targeted cetuximab treatment for patients with wild-type RAS mCRC yields an ICER of approximately $650,000 per LY in comparison with anti-EGFR therapy from the perspective of the United States [16]. One possible reason for differences in these estimates is that this study incorporated survival data derived from a different source, which resulted in a reduced survival benefit (0.0026 years) with the cetuximab regimen. In the National Cancer Institute of Canada trial CO.17, the addition of cetuximab produced an ICER and cost–utility ratio of $199,742 and $299,613, respectively. When cetuximab therapy was restricted to patients with wild-type RAS mCRC, the ICER was improved to $120,061 per LY gained and $186,761 per QALY gained [17, 21]. An economic analysis in Switzerland showed that the ICER of cetuximab treatment as last-line therapy for patients with mCRC was €62,653 per QALY gained compared with that of regimens without cetuximab, which indicates that gene-guided cetuximab treatment is economically favorable [22].

The potential of cetuximab, the first therapeutic antibody for mCRC, to improve survival is a major determinant of clinical and economic outcomes. One-way sensitivity analysis found that the median OS time of the cetuximab regimen was the most influential parameter. This result indicates that the selection of a patient subgroup can increase the cost-effectiveness of the addition of cetuximab. Other independent and influential parameters include health insurance coverage and the price of cetuximab. A higher proportion of coverage will lead to a higher ICER for the addition of cetuximab treatment. As a potential option, providing a more favorable discount or PAP plan for cetuximab would significantly decrease the ICER for the addition of cetuximab.

Several important limitations in the current study should be considered. First, modeling to extrapolate clinical survival beyond trial observation is an inevitable limitation in this study. The present model showed that PFS and OS time had substantial effects on the model’s outcome. The short median follow-up periods of the pivotal cetuximab trials did not provide enough observed survival data to compare with the median survival estimated by the model. Thus, there was much uncertainty in the long-term survival probability. Second, the model did not fully evaluate the outcomes of using cetuximab in other settings, such as extended RAS testing, sensitivity or specificity of the KRAS mutation-screening test, second- or third-line treatment and combination with other chemotherapy regimens, which should be investigated in the future. Third, the present model did not include other biologicals used as first-line chemotherapy drugs, such as panitumumab, for assessing the incremental cost-effectiveness in comparison with cetuximab, as these drugs have not been approved by the Chinese Food and Drug Administration. Fourth, we did not perform a budget impact analysis of the addition of cetuximab. The age-standardized mortality was 16.9 per 100,000 in males and 11.6 per 100,000 in females [2], and cetuximab might be prescribed to more than 10,000 patients each year. Based on the results from our model, the addition of cetuximab to standard chemotherapy will increase expenditures by approximately $131 million. Fifth, the clinical data were derived from trials from other countries, potentially influencing the results owing to radial differences. However, the Chinese study showed similar efficacy and safety to that found in a Caucasian population [23]. Sixth, the utility values obtained from other regions and the triangular distribution of cost inputs may have biased the model’s output. Finally, the current analysis did not assess the impact of different therapies after disease progression. However, the results of the one-way sensitivity analysis indicated that the costs of disease progression had little impact on the final results. Owing to these limitations, the results should be carefully explained when they are referenced by Chinese decision makers.

Our analysis indicates that the addition of cetuximab to traditional chemotherapy in patients with wild-type RAS mCRC is likely to be a cost-effective recommendation in China based on its superior efficacy and association with PAP. Although the current analysis focused on the Chinese medical system, the findings may also be helpful to other medium-income regions, such as Brazil, Russia, Taiwan and Thailand.

## MATERIALS AND METHODS

### Analytical overview and model structure

A mathematical model was established to measure clinical and economic outcomes of additional cetuximab therapy for patients with mCRC. Patients were assumed to either start standard chemotherapy based on irinotecan, fluorouracil, and leucovorin (FOLFIRI, control regimen) or to start targeted treatment with additional cetuximab if the RAS screening was negative (cetuximab regimen), as shown in Figure 3A. Because this chemotherapy has been recommended as the first-line standard treatment for newly diagnosed mCRC by clinical guidelines [11] and the aim was to evaluate the economic outcome of adding cetuximab to the standard chemotherapy regimen, a “no treatment” strategy was not evaluated in this study. Health and economic outcomes were predicted using the Markov state transition model (Figure 3B) with three exclusive health parameters: PFS, progressed survival and death. A hypothetical cohort with confirmed newly diagnosed mCRC was created for comparing cetuximab therapy with a control regimen. We set the characteristics of the hypothetical cohort to be similar to the phase III ARTIST trial, which showed the age of 214 Chinese patients with newly diagnosed mCRC was 53 years old (range: 23–77), proportion of male was 50.4% and proportions of primary tumor site in colon, rectum and colorectum were 47.5%, 47.5% and 5.0%, respectively [24]. After cancer progression, patients were treated with second-line chemotherapy or supportive care. The duration was ten years because the median OS of patients with mCRC was lower than 3 years and the probability of survival to year 6 was zero in the FIRE-3 trial. The Markov cycle length was 14 days, and the primary evaluation criterion for all patients was PFS. The risk of disease progression or death was determined by the reported literature [7, 25]. This economic analysis was based on a literature review and experimental model and did not require approval by the Institutional Review Board/Ethics Committee.

The following outcomes were examined: progression-free LYs, overall LYs, QALYs and cost. Cost and QALYs were annually discounted 5% based on the Chinese guidelines for pharmacoeconomic evaluation [26, 27]. The costs are shown as 2016 US dollars. ICERs presented as the cost per additional QALY gained were also examined.

### Clinical data

We carried out a literature review to identify all randomized controlled trials (RCT) exploring the clinical effectiveness of cetuximab in combination with FOLFIRI chemotherapy in comparison to FOLFIRI chemotherapy alone in patients with previously untreated mCRC. The following databases were used to search for eligible studies (cut-off date of March 26, 2016): PubMed, Web of science, EMBASE, and the Cochrane Library. The systematic searches identified two studies, which were included in the literature review of clinical effectiveness. Table 2 lists the key model parameters.

**Table 2:** Key model inputs

Parameter	Values(ranges)	Description and Reference
Weibull survival model of PFS of control regimen	Scale=0.00267; Shape=1.89552; r^2^=0.992	[7]
Weibull survival model of PFS of control regimen	Scale=0.00195; Shape=1.52888; r^2^=0.976	[7]
Weibull survival model of OS of cetuximab regimen	Scale=0.00540; Shape=1.53841; r^2^=0.982	[25]
Weibull survival model of OS of cetuximab regimen	Scale=0.00324; Shape=1.26410; r^2^=0.978	[25]
RAS mutation prevalence	0.41(0.366-0.454)	[28, 29]
Body surface (m^2^)	1.72 (1.5-1.9)	[37]
Cost of FOLFIRI per cycle (US $)	2050.5 (1083-3018)	[38, 39]
Cost of cetuximab per 100 mg (US $)	637.4 (318.7-637.4)	[40]
Cost of salvage therapy per cycle (US $)	2411.8 (1891-2739.1)	[38, 39]
Cost of RAS screening pre unit (US $)	176.9 (132.7-221.2)	[40]
Cost of terminal care per cycle (US $)	1980.1 (769.2-5288.3)	[37]
Cost of vomiting per event (US $)	175.7 (134-223)	[41–43]
Cost of rash and acne per event (US $)	11.1 (6.2-16)	[41–43]
Cost of fatigue per event (US $)	1524.6 (421.9-3322.6)	[41–43]
Cost of neutropenia per event (US $)	2694.6 (2154.7-3294)	[41–43]
Cost of diarrhea per event (US $)	891.5 (158.6-1104.6)	[41–43]
Utility of PFS	0.85 (0.68-1)	[36]
Utility of progressed survival in chemotherapy or supportive care	0.24 (0.2-0.28)	[35]
Utility of progressed survival in targeted therapy	0.68 (0.52-0.78)	[11, 34]

FOLFIRI: irinotecan, fluorouracil, and leucovorin; PFS: progression-free survival; OS, overall survival; RAS: rat sarcoma viral oncogene homolog.

Kaplan-Meier survival data of PFS and OS for the control regimen were available from the CRYSTAL trial, which evaluated the efficacy of 599 patients receiving FOLFIRI alone [7], and the clinical benefit of the cetuximab regimen was derived from the FIRE-3 study, which evaluated the efficacy of cetuximab plus FOLFIRI treatment in 297 patients with KRAS (exon 2) codon 12/13 wild-type mCRC [25]. The Weibull survival model was fitted to the reported PFS and OS survival data. Estimated scale and shape parameters, standard errors (SEs), adjusted R^2^ and correlation coefficients are presented in Table 2. The shape parameter (γ) allows the hazard function to increase or decrease with increasing time; if γ > 1.0, the hazard rate strictly increases in a nonlinear pattern with increasing time. The scale parameter (λ) is related to the measurement unit of time. It is assumed that RAS mutation status has no impact on the efficacy of FOLFIRI therapy [7]. The prevalence of mutations of KRAS was 41% in Chinese CRC patients [28, 29]. Tumor mutation status of KRAS was assessed using a pyrosequencing approach, as described in the FIRE-3 trial [25]. The current analysis assumed that there was no statistically significant difference in the sensitivity or specificity of the KRAS mutation-screening test in comparison with the FIRE-3 trial.

### Cost and utility

We used the Chinese medical insurance perspective to estimate the cost of direct health expenditures, including first-line study treatment and second-line chemotherapy due to disease progression, follow-up and other direct medical costs (Table 2). Treatment of side effects was considered only for SAEs (grade 3-4). All unit costs of health resources were obtained from the local literature, the health system or the National Development and Reform Commission of China. Catastrophic disease insurance would cover 60% of the medical expenditure [30–32].

Based on a cycle length of 14 days, the treatment scheme was as follows: FOLFIRI comprised a 60- to 90-min infusion of 180 mg/m^2^ irinotecan, a 120-min infusion of 400 mg/m^2^ racemic folinic acid, and 400 mg/m^2^ fluorouracil followed by a continuous 46-h infusion of 2,400 mg/m^2^ fluorouracil. The cetuximab regimen consisted of cetuximab (initial dose 400 mg/m^2^ infused over 120 min, and 250 mg/m^2^ infused over 60 min weekly thereafter) plus FOLFIRI. Treatment was continued until disease progression or unacceptable toxicity. Once the disease progressed, patients were assumed to receive salvage chemotherapy. To estimate the dosages of chemotherapeutic agents, it was assumed that a typical patient had a weight of 65 kg and a height of 1.64 m with a body surface area (BSA) of 1.72 m^2^, unused drugs in opened vials were discarded [33].

Because of the high price of cetuximab, it is not affordable by many in China; as such, the cetuximab PAP was implemented for Chinese patients with mCRC. In this program, cetuximab is paid for by the payer for the first two months, followed by donations for two months by the producer. Subsequently, cetuximab is supplied by the following scheme: pay for 1 month + donation for 3 months. Therefore, the impact of PAP was incorporated into the scenario analyses.

The utility scores of PFS and progressed survival were obtained from previously published studies (Table 2), and their standard errors were estimated at 25% of the mean value in our sensitivity analyses [11, 34–36].

### Sensitivity analyses

To test the robustness of the model, one-way sensitivity analyses and PSA were used. In the PSA, key model inputs were simultaneously and randomly sampled from the statistical distributions to generate 1,000 estimates of the cost and QALY for both regimens. Triangle distributions were adopted for cost parameters owing to the limited number of samples for generating the cost data, and the beta distribution was used for probability, proportion and utility score parameters. A cost-effectiveness acceptability curve (CEAC) was shown based on the results of the PSA. One-way sensitivity analyses were carried out for all parameters in a predefined range as shown in Table 2, which were mainly obtained from the reported literature or by assuming a 25% or 50% base-case value. The model was constructed and analyzed in the R statistical environment (version 3.2.3; R Development Core Team, Vienna, Austria). In accordance with the World Health Organization (WHO) recommendation [44–46], the 3× per capita gross domestic product (GDP) value of China in 2015 ($22,200) was used as the cost-effectiveness threshold.
